# Aptamers and Their Potential to Selectively Target Aspects of EGF, Wnt/β-Catenin and TGFβ–Smad Family Signaling

**DOI:** 10.3390/ijms14046690

**Published:** 2013-03-26

**Authors:** Andrea Conidi, Veronique van den Berghe, Danny Huylebroeck

**Affiliations:** Laboratory of Molecular Biology (Celgen), Department of Development and Regeneration, KU Leuven, Campus Gasthuisberg, Building Ond & Nav4 p.o.box 812, room 05.313, Stem Cell Institute, Herestraat 49, B-3000 Leuven, Belgium; E-Mails: andrea.conidi@med.kuleuven.be (A.C.); veronique.vandenberghe@med.kuleuven.be (V.B.)

**Keywords:** aptamers, β-catenin, EGF, signal transduction, Smad-interacting proteins, Smad, TGFβ, Wnt

## Abstract

The smooth identification and low-cost production of highly specific agents that interfere with signaling cascades by targeting an active domain in surface receptors, cytoplasmic and nuclear effector proteins, remain important challenges in biomedical research. We propose that peptide aptamers can provide a very useful and new alternative for interfering with protein–protein interactions in intracellular signal transduction cascades, including those emanating from activated receptors for growth factors. By their targeting of short, linear motif type of interactions, peptide aptamers have joined nucleic acid aptamers for use in signaling studies because of their ease of production, their stability, their high specificity and affinity for individual target proteins, and their use in high-throughput screening protocols. Furthermore, they are entering clinical trials for treatment of several complex, pathological conditions. Here, we present a brief survey of the use of aptamers in signaling pathways, in particular of polypeptide growth factors, starting with the published as well as potential applications of aptamers targeting Epidermal Growth Factor Receptor signaling. We then discuss the opportunities for using aptamers in other complex pathways, including Wnt/β-catenin, and focus on Transforming Growth Factor-β/Smad family signaling.

## 1. Aptamers: Emerging, but Re-Discovered Precious Tools in Diverse Studies

In 1990, Ellington and Szostak [1], in the context of their studies on self-splicing group I introns and ribozymes, reported on the production and characterization of short, random RNA sequences able to bind specifically to one of various organic dyes used as target. About one in ten billion random RNA sequences apparently folds in such a way that it creates specific binding to one of their small dyes. They called these RNA sequences—and later similarly behaving and selected single-stranded DNA molecules [2]—aptamers. Aptamers have meanwhile been divided in two types, *i.e.*, nucleic acid aptamers and peptide aptamers.

Nucleic acid aptamers are short, single-stranded RNAs and DNAs that represent high-affinity and highly selective ligands for different targets, ranging from large proteins to peptides, nucleotides, drugs and small compounds, and even metal ions. Such aptamers can be isolated from combinatorial libraries by SELEX (systematic evolution of ligands by exponential enrichment) [3]. The SELEX approach has been optimized over many years and used in different studies, including for targeting in cancer cell lines [4–6]. Briefly, a large library of high-complexity oligonucleotide sequences, comprised of a randomized region that is flanked by constant regions carrying annealing sites for primers in PCR-based amplification, is incubated with the target, e.g., a purified protein. Then, the unbound nucleic acids are separated from the bound ones. The latter are subsequently dissociated from the aptamer–protein complexes and amplified by PCR. Several rounds of selection, partition and amplification can be performed to obtain PCR products with higher affinity and specificity for the target. The eventual PCR products are then sequenced to identify the best binding sequences in the library whose complexity was reduced. Higher-order conformations, *i.e.*, structures such as stem-loops, are fundamental to the activity of nucleic acid aptamers. This structural feature depends on the primary sequence and its length (generally 15–100 nucleotides; the length co-determines the efficiency of delivery to cells, as well) and even the experimental conditions (e.g., in cases of metal ion-dependent drug interactions) [7,8]. The structure underlies also the stability of such aptamers and their binding to molecules ranging from small ions (such as Zn^2+^) [9] and small organic compounds (e.g., aminoglycoside antibiotics) [10] to large targets such as glycoproteins (e.g., CD4) [11]. SELEX was recently modified further in order to target surface proteins of intact, living cells (Cell-SELEX). Striking examples are Chinese Hamster Ovary (CHO) cells, forced to produce TGFβ type III receptor (TβRIII), used as a target for a screening of RNA aptamers against this receptor [12]. In a similar whole-cell SELEX study in PC12 cells, a neutralizing aptamer that blocked ligand-induced wild-type Ret receptor and also signaling of a dimerization-inducing activating mutant receptor has been isolated [13].

Peptide aptamers are combinatorial proteins consisting of a variable peptide inserted into a scaffold protein [14]. Conceptually, they can be compared to antibodies whose antigen-binding domains are formed by a variable and constant region held together by disulfide bonds. Unlike antibodies, peptide aptamers have a small size and a simple design, are in most cases independent of disulfide bonds, and seem to function well in living cells (see Table 1).

In addition, peptide aptamers are dually constrained because their *N*- and *C*-termini are fused to the scaffold. In general, constrained peptides possess several advantages compared to flexible peptides with free ends. First, even in the unbound state, the constrained peptide insert usually has higher affinity for its target than its flexible counterpart. For example, within the scaffold, the affinities of the variable peptide moieties to their target proteins decreased from 10 to 1000-fold when they were taken out from their scaffold and tested as such [15]. Second, peptides displayed within a scaffold often exhibit higher stability and protease resistance than linear peptides [16], which is relevant in case the peptide aptamers are used to target intracellular proteins. Third, mainly the early work in the context of bacteriophage display methodology showed also that constrained peptides have a reduced degree of conformational freedom [17].

Perhaps the most popular scaffold protein for displaying constrained peptides is Thioredoxin-A (TrxA) of *E. coli* (Table 2). It has been chosen because of its small size (about 12 kDa), high stability and solubility, and its known secondary and tertiary structure [16,18,19]. In addition, TrxA, like any other ideal scaffold protein, is also unrelated and inert to the physiology of eukaryote cells. TrxA is a globular protein with a catalytic domain that is solvent-exposed. Insertion of a peptide within this catalytic domain disrupts the TrxA enzymatic activity, but assures that the constrained peptides are exposed for interaction with their target.

Alternative scaffold proteins (see Table 2) include green fluorescent protein (GFP) for use in eukaryote cells [20], a catalytically inactive variant of staphylococcal nuclease (used in yeast) [21,22] and an optimized variant of stefin A, a protease inhibitor of cathepsins. Stefin A has been engineered to provide surface immobilization of the peptides as well as ensuring exposure of the binding site to the target solution, and avoiding any interactions with human proteins [23,24].

The action mechanisms underlying bioactive peptide aptamers (from here we use “peptide aptamer” as the combination of the scaffold protein with its peptide insert) have been investigated in different studies. In most of these, peptide aptamers have been shown or tested for inhibiting protein–protein interaction [15,18,19,28,29]. Many studies reported on selected aptamers that bind to transcription factors (TFs) and inhibit TF binding to DNA. The latter is achieved by masking the DNA-binding domain directly or by inhibiting a protein–protein interaction required for the TF to bind to DNA or for its transcriptional activity [30,31]. Another action mode by peptide aptamers may include sequestration of their target protein in inclusion bodies, called aggresomes, in the perinuclear region as shown for the hepatitis B virus core protein and the human papillomavirus E6 oncoprotein [32]. However, not all peptide aptamers inhibit target protein function(s). For example, Nouvion and co-workers produced peptide aptamers that target different regions of the anti-apoptotic protein Nr-13. Interestingly, they found that the majority of their aptamers could work as inhibitors of Nr-13 and thus promote apoptosis in cancer cells, but one aptamer directed against (another region of) Nr-13 worked as agonist, thus protecting cells from apoptosis [33]. In another study, involving a screening for aptamers (in a lentiviral-based library) that inhibit cell proliferation *in vitro*, the selected positive aptamer R5G42 was subsequently used as bait in a yeast two-hybrid screening and identified Calcineurin, known to activate lymphocytes and is, in fact, a protein phosphatase, *i.e.*, of Tau, linked to Alzheimer’s disease, and of BAD, a member of the Bcl2 family of apoptosis regulator factors, where BAD–Bcl2 binding has been documented. R5G42 binds to and activates the Calcineurin phosphatase activity and hence provokes dephosphorylation of BAD, and even can re-establish the effects on proliferation in cultured cells treated with inhibitors of Calcineurin [34].

Collectively, these studies illustrate that aptamer-induced perturbations can resemble those caused by small molecules, including some small molecules that are already used in therapy. These results make peptide aptamers useful tools in discovery of small molecule drugs, due to the ability of aptamers to bind to various polymorphic protein surfaces. When the binding site of peptide aptamers is mapped on their target protein, structural studies can subsequently be performed on these surfaces. In most cases, the latter will turn out “druggable,” meaning that they are accessible to small molecules and two complementary approaches can then be followed. In the first approach, a virtual screening for target-based pharmacological designs can be performed against these surfaces. In the second approach, the aptamer can be used in high-throughput screening protocols to identify small molecules that disrupt the interaction between target protein and peptide aptamer [35].

## 2. From Bench to Bedside: Aptamers Enter Diagnostics and Therapy

In recent years aptamers have also moved from basic research (see section 1) to the clinic. Concerning their use in therapy, a major breakthrough was the approval for using Pegaptanib/Macugen©, a RNA aptamer directed against VEGF165 (for a review on evolutions in aptamer therapeutics, see [36]). This aptamer is used to treat age-related macular degeneration (AMD), as well as cases of diabetic macular edema (DME) (Table 3). These retinopathies are accompanied by increased VEGF-based vascularization of the retina, causing blurry vision, myopia, and, in most severe cases, blindness. Macugen was the first aptamer to enter clinical trials and obtain FDA approval in 2005 for treatment of retinopathies [37]. We could trace about 20 ongoing aptamer-based clinical trials at present (http://clinicaltrials.gov/ct2/results?term=aptamer; see Table 3; for a recent review of the state of the art in e.g., RNA aptamer-based clinical trials, see [38]).

Amongst these, of particular interest are the ones using nucleic aptamers that (i) target platelet-derived growth factor (PDGF) for inducing neovascular regression in AMD (E10030 aptamer); (ii) modulate hemostasis, via targeting selected factors of the coagulation pathway, thus making such aptamers new anti-coagulants for e.g., post-surgery interventions (aptamers NU172, RB006 and ARC1179) or for treatment of hemophilia (aptamer ARC19499); (iii) provide a treatment for diabetes mellitus (aptamer NOX-E36); and (iv) specifically target tumor cells, as in the case of the AS1411 aptamer, which binds to nucleolin, a protein overproduced by cancer cells, and that plays a role in promoting cell survival and proliferation. AS1411 is currently in an advanced Phase 2 stage for treatment of acute myeloid leukemia (AML) (see Table 3), but also of renal cell carcinoma. Overall, such aptamers can be administered intravenously, subcutaneously or intravitreally, with the intravenous route being more efficient, for the aptamers seem to be fully bioavailable in that case (for a discussion, see [39]).

Prior to the approval for use in any clinical trial, the action mechanism, specificity and affinity of each aptamer for the target protein have to be characterized, together with the aptamer toxicity and the aptamer pharmacokinetics. Nucleic acid aptamers like the ones currently in clinical trial are not stable *per se* but sometimes require chemical modifications for increasing their resistance to endonucleases, increasing their half-life, as well as controlling their renal clearance (reviewed in [38–40]).

Aptamers are recent new tools in the clinic and there is no extensive literature yet on their collateral effects, including in preclinical safety assessment studies, which have been conducted in different animal species (rodents, monkeys), using single *versus* repeated doses. Toxicological and pharmacological information has been reported on antisense oligonucleotides (ASOs). The conclusions are, in the latter case, that three main effects should be carefully considered when designing ASOs or other oligonucleotides for drug purposes, *i.e.*, the polyanion effect, the tissue accumulation of the oligonucleotides, and their stimulation of innate immunity, respectively (for a recent review, see [39]). The polyanion effect of oligonucleotides is caused by sequence-independent, unintended (or off-target) protein interactions. For a shorter therapeutic aptamer, which is selected and optimized for specific and high-affinity interaction and for use at optimal concentrations, this is likely a lower risk factor. In general, studies of the effects of aptamers (including the PEGylated aptamers) when used at optimal concentrations—on complement C′ activation and connected anti-coagulation, and on bone marrow suppression, hemostasis (and hemodilution), respiratory performance, and genetic toxicity, show that they present with a good safety margin, which in the worst case equals the margin documented for other oligonucleotides or (PEGylated) macromolecules. Stimulations of innate immunity, observed in rodents, were initially associated with CpG motifs within single-stranded DNA and a specific interaction with Toll-Like Receptor 9 (TLR9). Further studies have shown that also RNA-based molecules can stimulate other TLRs (e.g., TLRs 3, 7 and 8) and elicit activation of innate immune responses, which result in hyperplasia of lymphoid organs and infiltration of mononuclear cells in non-lymphoid organs.

## 3. Aptamers and Their Use in Targeting of Signal Transduction Cascades

Advances in aptamer chemistry and optimized ways of delivery of aptamers into cells cleared the way to use peptide aptamers as candidate therapeutic molecules against intracellular targets, including effector proteins of growth factor receptor initiated signaling. We have selected examples of the application of aptamers in two intensively studied signaling cascades relevant to embryogenesis, as well as cancers of different histo-pathological classes, *i.e.*, epidermal growth factor (EGF) and Wnt signaling. Thereafter, we will focus on TGFβ family signaling.

### 3.1. EGF Signaling

In EGF signaling, peptide aptamers have been selected based on their ability to bind to the intracytoplasmic domain of the EGF receptor (EGFR) or to the downstream TF Stat3. Sustained activation of EGF family receptors (in vertebrates, ErbB1 to ErbB4; for a review, see [41]) is a common hallmark of several types of cancer, in particular breast, lung, ovarian and gastrointestinal cancers. These tyrosine kinase receptors, one of which is almost devoid of kinase activity (ErbB3) and another one (ErbB2, also named Her2) not binding to any known EGF-like ligand, can homo/hetero-dimerize to form stable transmembrane complexes with different affinities for specific EGF family ligands, including EGF itself, TGFα, amphiregulin and neuregulin. Mutation or overexpression of EGFRs (well documented for ErbB1 and ErbB2), including through gene amplification (e.g., for *HER2*/*ERBB2*) are often associated with a poor prognosis in cancer patients. ErbB2 is considered a positive regulator (via amplifying and prolonging) of signaling, partly based on its deviant biochemistry, e.g., binding a larger set of phospho-Tyr binding proteins than the other ErbB receptors, and its over-activity directly correlates with poor prognosis. Activating mutations in the receptor kinase domain can also result in ligand-independent signaling.

**Figure 1:**
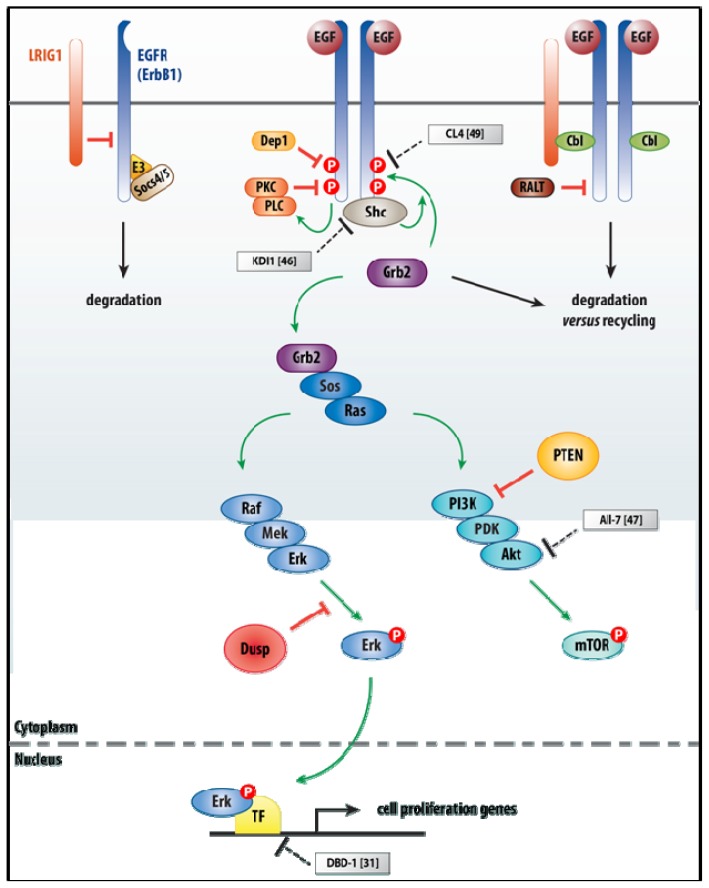
An overview of signaling by the EGF family (with EGF and EGFR/ErbB1 as example) from cell surface down to the nucleus (without taking into account endocytic trafficking and recycling of the receptor). In the absence of ligand (left panel), LRIG1, a negative regulator (red line), binds to the EGFR and both are degraded by less well-studied mechanisms. In addition, unliganded EGFR can also be sent into E3 ubiquitin ligase-Socs4/5-assisted ubiquitylation and degradation. In the presence of ligand (middle and right panel), still two outcomes are possible. In the first, positive signaling (green arrows) is initiated via Grb2, resulting in activation of downstream kinase cascades Raf–Mek–Erk (Erk or Mapk) and PI3K–PDK–Akt, which is a prototypic survival pathway that is constitutively activated in many types of cancer. The latter cascades are also subject to negative regulation exerted by dual-specificity phosphatases (Dusp) and the tumor-suppressor PTEN, respectively. In the nucleus, Erk teams up with an activated TF to induce transcription of cell proliferation genes. In the second, degradation *versus* recycling is still an option, one initiated via Grb2 and one—also in a ligand-dependent manner—where LRIG1-Cbl (Cbl is an E3 ubiquitin–protein ligase) and probably direct EGFR–Cbl interaction ensure full ubiquitylation and hence degradation of the EGFRs. Finally, also RALT, a pan-ErbB inhibitor, acts at this level: it inhibits EGFR allosteric kinase activation. Grey boxes highlight the aptamers discussed in the main text, together with the references.

Ligand binding to the ectodomain of the receptor chains induces a conformational change in the receptor within the receptor complex, resulting in auto-phosphorylation of the respective intracytoplasmic domains in the receptor complex. These phospho-Tyr residues enable docking of several cytoplasmic signaling proteins and activation of signaling via Sos to Ras, and then via Raf–Mek–Erk and PI3K–PDK–AKT pathways (see Figure 1). Thus, the system amplifies the signal and activates other proliferation-regulatory proteins (Ras), but also anti-apoptotic proteins (Bcl2), cytoskeleton regulatory proteins (hMena(11a)) and TFs (in particular Stat3), leading to e.g., the promotion of cell survival and migration [42–44]. In addition, like in most growth factor receptor families, the EGFR system is controlled by feedback and feedforward loops (for a review, see [45]), by spatial–temporal control of signaling, for example, at the level of endocytic trafficking of the ligand–receptor complexes, but also including escape of the receptor heterodimers from Cbl leading to their recycling rather than degradation (the latter being the case for homodimers). In addition, the system is downregulated also by Tyr-specific phosphatases.

Administration of anti-EGF or optimized anti-Her2/ErbB2 monoclonal antibody (Herceptin/Trastuzumab) are the most widely used—but also very expensive—inhibitors of EGF signaling for the treatment of cancer. Furthermore, such therapeutic protocols can still coincide with severe collateral side effects. Trx-based peptide aptamers against EGFR that complex with its intracytoplasmic domain, and that provoke slower proliferation in a dose-dependent fashion and reduce soft agar colony formation by the tumor cells, have been obtained. Specifically, the KDI1 aptamer does not block the the receptor’s kinase activity, but affects the activation of downstream p46 Shc and Stat3, including the transcription activation by the latter [46]. In addition, peptide aptamers specifically designed to bind the dimerization domain or the DNA-binding domain within Stat3 can abolish Stat3 DNA-binding and hence transcriptional activation by Stat3 in EGF-stimulated cells. In myeloma cells these same aptamers cause growth inhibition, downregulation of Bcl family members and induction of apoptosis [31]. Peptide aptamers were selected also to target the ErbB2 kinase domain. In this case, the peptide aptamer AII-7 resulted in modest reduction of the activation of Stat3, had no effect on downstream p42/44 MAP kinase activation, but inhibits Akt kinase in MCF7 breast cancer cells treated with heregulin. This is relevant, for sustained activation of Akt is responsible for the increased resistance of ErbB2-overproducing tumors towards chemotherapy. Altogether, AII-7 restored the sensitivity of breast cancer cells toward the mitosis inhibitor Paclitaxel/Taxol [47]. Notably, in each of these examples a new approach in the production of the peptide aptamers was used. Indeed, a protein transduction domain (PTD) was fused to the recombinant protein, increasing its uptake by cells [48]. The most frequently used PTD is derived from human immunodeficiency virus (HIV-1) Tat protein.

More recently, a nuclease-resistant RNA-aptamer, named CL4, was produced based on SELEX [49]. It specifically binds the EGFR with high affinity (*K*_d_: 10^−9^ M), whereas it does not bind to other ErbB-family members. EGFR-overproducing cells treated with CL4 show a block in EGFR-mediated signaling and induce a cell death program. At low doses, this aptamer is able to induce apoptosis even in cells that are resistant to some of the commonly used EGFR inhibitors (e.g., Cetuximab and Gefitinib). It also slows down tumor growth in mice xenografted with human non-small-cell lung cancer (NSCLC) cells. CL4 can also induce apoptosis in EGFR-producing cells in a synergistic manner with Cetuximab [49]. Thus, CL4 represents a novel, more efficient approach to target EGFR signaling compared to the classic inhibitors. Further studies of its toxicity, half-life and renal clearance are required to take this aptamer into potential clinical therapies.

### 3.2. Wnt/β-Catenin Signaling

**Figure 2:**
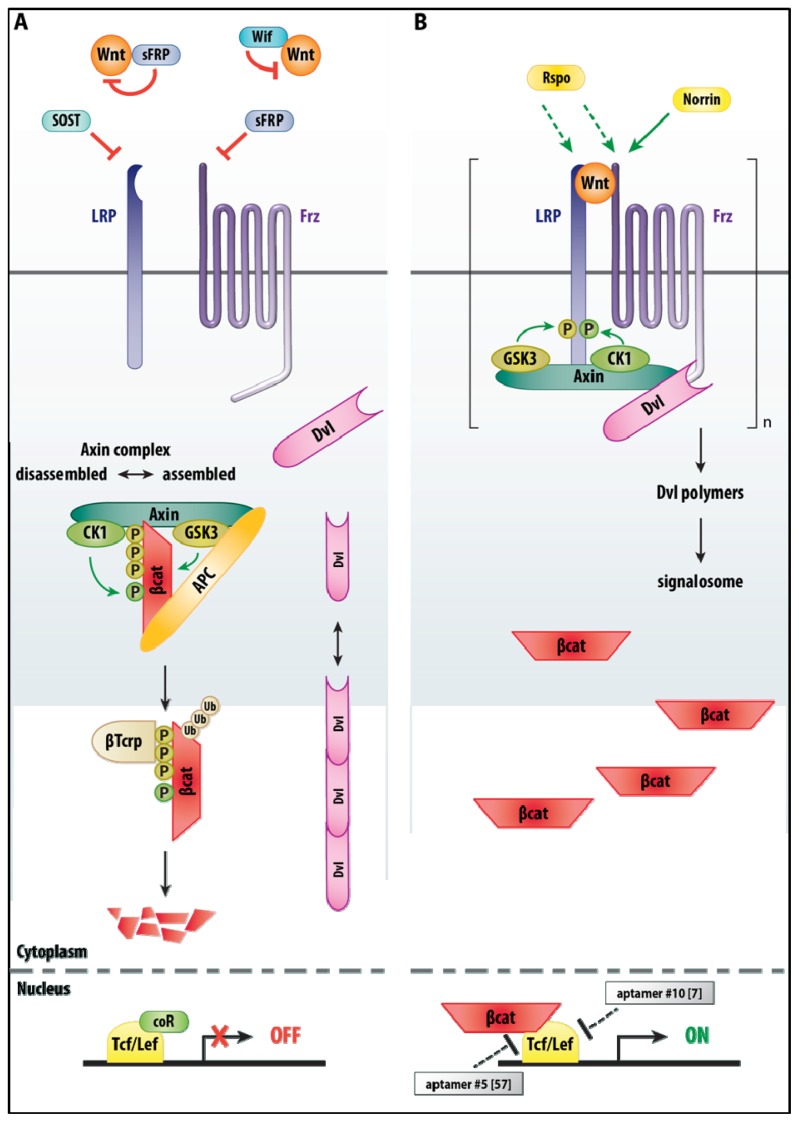
A compilation of previous with revised Wnt-β-catenin (β-cat) signaling in the absence (panel A) and presence of bio-active Wnt (panel B), from cell surface down to the nucleus, without taking into account endocytic trafficking or more detailed cytoplasmic sublocalization of the components. Red lines indicate inhibitory actions by e.g., secreted antagonists (sFRP, Sost, WIF), green arrows represent stimulatory actions by extracellular agonists (Rspo, Norrin) or phosphorylation exerted by the kinases GSK3 and CK1. [ ]_n_ in the panel to the right indicates the formation of a clustered LRP–Wnt–Frz signaling platform (signalosome) via increased polymeric state of Dvl; also clustering of multiple LRP6 proteins with a LRP–Wnt–Frz complex has been reported (not shown in panel B). Ub, ubiquitylation of β-cat; coR, co-repressor; ↔, equilibrium; P, phosphorylated residues. For further details, see main text. Grey boxes highlight the aptamers discussed in the main text, together with the references.

The Wnt family of secreted (glycolipo)protein ligands are critical in embryonic development, in adult stem/progenitor cells and during regeneration after acute injury, where in particular the “canonical” signaling (implicating β-catenin) regulates cell fate, cell proliferation and stem cell self-renewal [50]. The Wnt ligands bind to a receptor complex composed of members of two distinct receptor families, *i.e.*, the large family of Frizzled (Frz) seven-pass transmembrane receptors and two members of the LDL receptor-related protein family (*i.e.*, LRP5 and LRP6) (Figure 2). However, the ligand-receptor system is more complex. For example, non-Frizzled receptors for Wnt ligands (such as Ror2, Cam-1, Drl, Ryk and Lin-18) have been identified (these were not included in Figure 2). In addition, secreted Frz-related proteins (SFRPs) and other proteins (like Wif) bind to Wnts, as well, and function as antagonists/modulators for both β-catenin and non-canonical signaling. Furthermore, two families of LRP5/6 ligand (of the Dkk and Wise/SOST families, respectively) are considered antagonists of Wnt/β-catenin signaling, whereas secreted R-spondin (Rspo) and Norrin proteins are two agonists (Figure 2) (for a review, see [51]).

The key co-operation between the receptors, for which most data in cell culture support the model wherein Wnt induces the formation of the Frz–LRP complex, is essential to keep Wnt signaling within homeostatic range [52]. In the absence of Wnt, intracellular Axin in the Axin multi-protein complex uses separate domains for interacting with the kinases GSK3 and CK1α, and with β-catenin. Axin coordinates the initial phosphorylation of β-catenin at Ser45 by CK1α and, subsequently, at Thr41, Ser37 and Ser33 by GSK3, with the two latter phospho-Ser enabling binding of the E3 ubiquitin ligase β-Trcp, causing degradation of β-catenin. Axin also contains a RGS (regulator of G protein signaling) domain that interacts with adenomatous polyposis coli (APC), a large multi-functional scaffold that itself binds β-catenin. In a revised model of Wnt signaling, the protein Dishevelled (Dvl), previously considered as the inactivator of the Axin destruction complex when Wnt was added, is proposed to reach equilibrium between its diffuse monomer state and its polymeric state in the cytoplasm in cells without added Wnt [53], still guaranteeing that β-catenin is degraded. In the presence of Wnt, the Wnt-induced LRP6 phosphorylation is a key event in Wnt receptor complex activation. LRP5/6 have five (Pro)_3_-Ser-Pro-X-Ser motifs, which represent docking sites for the Axin complex, thereby recruiting Axin to LRP5/6. Remarkably, the kinases that phosphorylate this motif are (first) GSK3 and (then) CK1. Thus, the phosphorylation of this motif is reversed—in terms of sequential order—compared to β-catenin phosphorylation, but primes the requirement and functionality of the same GSK3–Axin complex. Hence, the same kinase combination is used for positive as well as negative regulation of signaling.

In one more detailed model, recruitment of the Axin–GSK3 complex by Frz–Dvl initiates LRP6 phosphorylation by GSK3. Dvl and Axin would then form aggregates (via the so-called DIX domain in Dvl) that facilitate protein interactions that are weak and dynamic. This would result in Dvl-mediated receptor clustering and formation of a Wnt-loaded Frz-LRP-Axin-Dvl-GSK3-CK1 “signalosome” platform (see Figure 2). However, it cannot be excluded that Wnt would also cause LRP6 clustering and phosphorylation on itself [51] (not included in Figure 2). In any case, in the signalosome platform, plasma-membrane recruited Dvl polymers serve as scaffolds for Axin recruitment and inactivation, whereby β-catenin accumulates in the cytoplasm and subsequently—in the nucleus—participates in downstream gene transcription by Tcf/Lef TFs. The direct target genes for Wnt signaling are normally kept silent by an inhibitory complex of gene regulatory proteins, which include Lef/Tcf TFs bound to the co-repressor Groucho. In the nucleus β-catenin displaces Groucho and binds to Lef/Tcf (for a recent review on Wnt signaling and cancer-causing mutations in this pathway, see [50]).

Targeting the Wnt pathway for therapeutic purposes, despite its conceptually straightforward and linearity towards β-catenin and Tcf-1/Lef-1 in canonical signaling, is not an easy task seen in the complex regulations that were identified recently (see above). Valid recent approaches may be those that lead to an increase in the stability of Axin, thus promoting desphosphorylation of β-catenin. Axin is, for example, stabilized by ADP-ribosylation, which is mediated by Tankyrase enzymes. Targeting of Tankyrase-1 and/or -2 with the chemical XAV939 inhibitor increases Axin levels [54]. A well-suited target for inhibition could be the β-catenin/Tcf complex and its transcription. Tcf-1 binds via its *N*-terminal segment to a region encompassing 12 copies of 42 amino acids each (named Armadillo repeats) present within β-catenin. These 12 repeats form a highly closed “superhelix” that, together with the binding site of Tcf-1, form a large, positively charged groove that cannot easily be displaced by chemical compounds or altered by mutagenesis [55]. Small molecules (*i.e.*, PKF115-854 and CGP049090) have been produced that inhibit or disrupt β-catenin/Tcf-1 binding, thus opening new ways to tackle protein–protein interaction in Wnt signaling, but the specificity and affinity for this target complex remain to be precisely documented [56].

Jeong and co-workers [7,57] were the first to suggest the selection and use of RNA aptamers that impair β-catenin/Tcf-1 function. In a first study, such an aptamer was selected and found to bind to the *N*-terminal β-catenin-interacting domain of Tcf-1, affecting the binding of Tcf-1 to β-catenin and hence inhibiting their complex formation [57]. Another, simultaneous study of the same team describes a RNA aptamer that targets the DNA-binding domain of Tcf-1 and inhibits the interaction of endogenous Tcf-1 with its target DNA [7]. Interestingly, the authors describe intensively the structures of these RNA aptamers, which helps in understanding which structure is the most suitable to target Tcf-1. Even though further studies are required in order to improve the affinity of such RNA aptamers for Tcf/Lef-type factors in Wnt signaling, this approach has opened new avenues for interfering with Wnt signaling [58], especially when signaling levels exceed the homeostatic range and diseases (cancer) can result from this. Conversely, and possibly of interest for future applications, it is also known that levels that are too low may be behind degenerative conditions [52].

### 3.3. TGFβ Signaling

**Figure 3:**
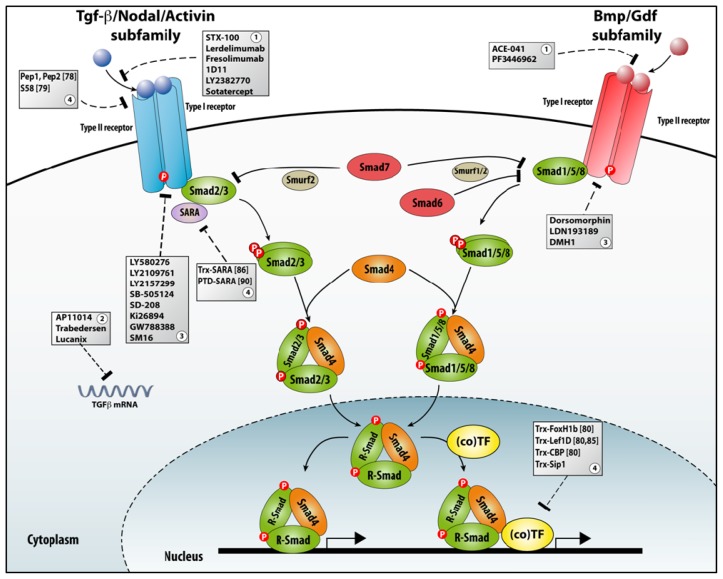
Schematic and simplified representation of TGFβ signaling, including negative regulations. TGFβ/Bmp ligand dimers bind to the respective type I and type II receptor-containing complexes, leading to activating phosphorylation (P) the intracytoplasmic domain of type I receptors. This type I receptor phosphorylates R-Smad proteins at two Ser residues at their C-terminus (see text) and enables their formation of a complex with the co-Smad Smad4. Inhibitory Smads (Smad6 and Smad7) interact with E3-ubiquitin ligases Smurf1/2 abolishing Smad signaling. Smad-anchor for receptor activation (SARA) protein is associated with the type I receptor and promotes interaction of the latter with Smad2/3. The Smad complex translocates and accumulates in the nucleus where Smads can bind to DNA via their MH1 domain and/or or interact with TFs or co-factors thereof, in a context of chromatin modulatory complexes (not shown). Grey boxes present different modes of interference with signaling, *i.e.*, ligand traps (**1**), antisense approaches (**2**), small molecule inhibitors (**3**) and peptide aptamers (**4**), respectively (for more details, see main text).

TGFβ family signaling controls cell proliferation *versus* differentiation, survival *versus* apoptosis, cell migration and shape changes, and cell adhesion to other cells and extracellular matrix. Deregulation of this signaling system leads to disease, e.g., fibrosis, cancer progression and metastasis, and disorders of multiple systems, including the immune system [59–61]. The TGFβ system is also crucial for embryogenesis and for the regulation of stem cells and their niche [61]. Signaling by this ligand-receptor system and its subsequent signal transduction are controlled at multiple subcellular levels, and the eventual outcome is dependent on cellular context (for a recent discussion, see [62]). The activation of the pathway starts with the ligand driving the assembly of a liganded tetrameric receptor complex [63–65] formed of two type I and two type II receptors, both with Ser/Thr-kinase activity in their intracytoplasmic domain. Both receptor types are needed in the signaling complex and the type I receptor in the complex determines the specificity of the activation of the downstream Smad proteins [61]. In the liganded receptor complex, the type II receptors phosphorylate the type I receptor in a GS-rich short sequence (also named the type I box) in its intracytoplasmic domain. The activated type I receptor becomes then docking site for effector proteins of the receptor-regulated Smad (R-Smad) family that are phosphorylated at two Ser residues located near to their C-terminus. The phospho–Smads then form a complex with Smad4. This Smad complex accumulates in the nucleus where it can bind to DNA with low affinity and/or to TFs, regulating target gene expression (Figure 3). Also non-Smad signaling, via a number of well-studied kinases, takes place (not included in Figure 3; for recent surveys, see [66,67]). Despite the strong convergence towards Smads, the pathway is remarkably diverse and complex, both upstream and downstream of the activated Smads, because of the many receptor-interacting and Smad-interacting proteins (SIPs; see below, section 4) that have been identified.

#### 3.3.1. Different Aspects of TGFβ Signaling in Pathological Conditions

TGFβs themselves, like other members of the family such as bone morphogenetic proteins (BMPs), are multi-potent cytokines involved in many cellular processes. In normal, non-transformed cells, and depending on cell type and context, TGFβ can function as an inducer of apoptosis, control cell proliferation, and strongly induce in epithelial cells a transition towards mesenchymal cells. TGFβ is also implicated in many aspects of tumorigenesis by directly acting on cells of the tumor as well as influencing their environment. During early stages of tumorigenesis TGFβ acts as a tumor suppressor, but at later stages it promotes tumor growth and enhances tumor invasion and metastasis, tumor-induced angiogenesis, and systemic and local tumor immunosuppression.

Aberrant TGFβ signaling causes diseases other than cancer. In hereditary hemorrhagic telangiectasia (HHT; see [68]) mutations have been identified in the genes for two receptors of the TGFβ/BMP family, *i.e.*, the co-receptor endoglin (for TGFβ, and BMP9 and BMP10; [69]) and the type I receptor ALK1 (for TGFβ and BMP; [70]), causing HHT often referred to as type-1 and type-2, respectively. Endoglin may also be insufficiently produced, may be produced as a truncated form that blocks TGFβ signaling, or inactivating somatic mutations may prevent endoglin production at lesion sites. TGFβ mRNA/protein levels have been documented in patients with diseases like kidney fibrosis and other nephropathies (for a survey, see [71]). Marfan’s syndrome is a disorder of connective tissue caused by mutations in the *FBN1* gene, which encodes fibrillin-1. Marfan patients show a severe deficiency in fibrillin-1 that would normally steer the formation and homeostasis of elastic fibers. The disease-associated manifestations of aortic aneurysm, pulmonary emphysema and eye lens dislocation were proposed to reflect structural weakness of the tissues. However, other aspects of Marfan’s syndrome (such as bone overgrowth and muscle hypoplasia) cannot be explained by this. Marfan-related disorders also exist, including some in which mutations in TGFβ family system components have been identified [72,73]. Despite the multiple functions of TGFβ, its blocking of or the interference with its signaling components remains an important, but still challenging, option in new therapies for the future [74].

#### 3.3.2. Many Roads to TGFβ Signaling Downregulation

Many antagonists of TGFβ signalling are being developed and are at a pre-clinical or clinical stage (Figure 3). The major classes include ligand traps (including antibodies), antisense oligonucleotides (ASO), small molecule receptor kinase inhibitors, and peptide aptamers. Ligand traps sequester TGFβ family ligands produced by tumor cells during cancer progression; a recent example is Sotatercept, a soluble activin type II receptor (ActRII-A) IgG-Fc fusion protein [75]. This class also includes ligand-neutralizing antibodies, as well as other soluble decoy receptor ectodomains (e.g., from either TβRII or TβRIII/β-glycan protein). ASOs are single-stranded, short molecules that hybridize to their complement RNA, inhibit mRNA function, and prevent target protein synthesis through accelerated mRNA degradation by RNaseH [76]. Small molecule receptor kinase inhibitors act via ATP-competitive inhibition of the catalytic activity within the receptor. Increased efforts have been initiated towards inhibition of BMP receptor signaling activities, relevant to a multitude of tissue repair and pathologic processes in the adult, using small molecule compounds [77]. Targeting intracellular proteins, such as Smads, via small peptides became the newest method and are therefore still least explored as a therapeutic strategy. We suggest that peptide aptamers will be ideal new tools to selectively inhibit a specific Smad activity based on their interaction with (one of) many SIPs, rather than hitting by receptor-targeting drugs (and in particular with the kinase inhibitors) the entire Smad signaling pathway. However, still few peptide aptamers have been designed to compete for the binding of some SIPs with Smad proteins.

#### 3.3.3. Aptamers as a Tool for Studying TGFβ Signaling

One of the most successful approaches to pharmacological inhibition of TGFβ signaling is to use monoclonal antibodies with high affinity for the receptors. Li and co-workers, using phage display, identified two peptides that bind to the ectodomain of both TβRI (Alk5), but also to TβRII, with similar affinity (*K*_d_ : 10^−5^ M). These peptides seem to prevent receptor complex assembly rather than occupying the two ligand-binding sites in the ligand-induced tetrameric receptor complex. Hence, the activation of an acknowledged TGFβ-Smad reporter transgene (based on the reiterated Smad-binding element (SBE) CAGA, *i.e.*, SBE_12_) in TGFβ-stimulated target cells was not affected. Neither of the two peptides could bind to e.g., BMPR–IA (Alk3), ActRII (which also binds BMPs) and the co-receptors β-glycan and endoglin [78]. More recently, aptamers were raised (using SELEX) against the extracellular domain of TβRII and used *in vitro* to investigate the TGFβ-induced myofibroblast transdifferentiation of human Tenon’s fibroblasts [79]. In particular, the aptamer S58 was found to antagonize the effects of TGFβ2 on differentiation, as the aptamer interferes with activation and nuclear translocation of Smad2. Transdifferentiated myofibroblasts show increased contractile activity associated with *de novo* expression of α-Smooth Muslce Actin (α-SMA), which is downregulated by S58 [79].

Cui and co-workers [80] produced TrxA-based peptide aptamers containing the respective Smad-binding domains (SBDs) of the SIPs FoxH1, Lef-1 or CBP. FoxH1 was the first nuclear SIP ever identified; this SIP-TF binds to specific conserved DNA sequences present in the promoter of several genes relevant to gastrulation. As mentioned above, their second selected target, Lef-1, binds nuclear β-catenin in Wnt signaling (section 3.2). Wnt and TGFβ family signaling co-operate in gene activation in early vertebrate embryos and in adult tissues. However, TGFβ-induced activation of target genes for Lef-1 is β-catenin independent, while it requires both Smad and Lef-1/Tcf-binding elements (TBEs) in the target promoters. Lef-1 turns out to be a SIP that interacts with Smad2/3 and Smad4 via two separate binding sites in the *C*-terminal HMG domain of Lef-1, *i.e.*, the basic region and the α-helix2, respectively [81]. CREB binding protein (CBP) and P300 are transcriptional co-activators that bind to many proteins (for a review, see [82]). They possess acetyltransferase activity mediating chromatin remodeling and the bridging of TFs to the transcriptional machinery. Inhibition of CBP is known to significantly reduce TGFβ transcriptional responses using several reporters. CBP is a SIP that binds to the MH2 domain of Smad2/3 via a *C*-terminal segment in CBP [83,84].

Cui and colleagues [80] produced a panel of different peptide TrxA-aptamers covering the Smad Binding Domains (SBDs) of these proteins FoxH1, LEF-1 and CBP, and they selected the ones presenting higher binding affinity for Smad3. They also tested whether such aptamers can bind to other Smads. FoxH1 and CBP aptamers were found to bind only to Smad2/3, whereas the Lef-1 aptamer (Lef-1D) binds to Smad4, Smad1, Smad2/3 and the inhibitory Smad (I-Smad) Smad7.

The produced set of aptamers was used in luciferase (lux)-based, TGFβ-sensitive promoter–reporter assays. The FoxH1 aptamer inhibits the A3-lux reporter, which contains three copies of an activin-responsive element. The Lef-1 aptamer inhibits TwntTop, a dual reporter of Smad signaling (via 2 SBEs) and Lef-1/Tcf (via 3 TBEs). In a second series of reporter assays (based on SBE_12_, TGFβ-responsive elements of the genes *PAI-1*, *Smad7* and *p15*, and the synthetic reporter 3TP-lux), only the FoxH1 peptide aptamer was able to inhibit 3TP-lux. Focusing on the Trx-based FoxH1 and Lef-1D peptide aptamers, the authors concluded that the Smads in the Smad complex expose different sites for the SBD of FoxH1 and Lef-1, and—importantly—that the binding of one aptamer to one site of the Smad target does not influence the binding to the second site.

Transfection of an expression construct for the Trx-based Lef-1D aptamer slows down cell proliferation, even regardless of stimulation of the cells with TGFβ [80]. In a follow-up study, it was found that expression of this aptamer reduced the expression of *c-Myc* via a mechanism involving binding of Smad4 to the *c-Myc* gene promoter [85]. TrxLef-1D aptamer expression resulted in a reduced activation of *c-Myc*. The *c-Myc* promoter has three TBEs, the first of which (TBE1) is bound by Smad4, which triggers *c-Myc* transcription in the absence of TGFβ. Upon addition of TGFβ, Smad4 dissociates from TBE1 and *c-Myc* is no longer transcribed. Thus, Smad4 can work in a TGFβ-independent fashion in *c-Myc* transcription, and the TrxLef-1D aptamer is able to block this. This makes Smad4-Lef1 interaction a potential therapeutic target when aiming at reduced proliferation of tumor cells.

The Hoffmann team also produced Trx-based peptide aptamers based on the SBD of SARA [86]. SARA (Smad anchor for receptor activation; also named Zfyve9) binds to Smad2 and Smad3 (the TGFβ/Activin/Nodal Smads) and facilitates their interaction with the intracytoplasmic domain of the type I receptor [87]). A similar protein (endofin; Zfyve16) has been proposed for the BMP–Smads Smad1/5/8) [88]. SARA binds Smad2/3 via a well-defined SBD consisting of a rigid coil, an α-helix and a β-strand, with each of these three elements being necessary for SARA–Smad interaction [89]. Only the SARA aptamer containing the entire SBD is able to bind to Smad2/3. This SARA aptamer binds only to TGFβ–Smads, but not to BMP–Smads, Smad4 and Smad7. Production of Trx–SARA aptamer impaired activation of Smad2/3, leading to diminished formation of the corresponding R-Smad–Smad4 complexes upon addition of the ligand. Interestingly, a total inhibition of TGFβ-induced epithelial-to-mesenchymal (EMT) transition was obtained using the SARA aptamer in cultures of normal murine mammary gland epithelial cells [86]. Recently, another group produced peptide aptamers where the SBD of SARA was linked to a PTD, resulting in an increase in peptide aptamer uptake by HK2 cells, a kidney tubule epithelial cell line. The authors observed a very slight increase in E-cadherin levels and a parallel reduction in -SMA expression when cells were transfected with the PTD–SARA aptamer and treated with TGFβ. They also report reduced Smad3 *C*-terminal phophorylation in PTD-SARA transfected cells after stimulation with TGFβ [90].

## 4. A Growing Number of SIPs Provide New Potential Aptamers for Interfering with Smad Signaling

The main difficulties in targeting TGFβ family signaling, which refrained pharmaceutical companies for a long time from entering this field, are its multiple effects, the strong convergence to Smad signaling, and the dual, opposite effects it can exert together with the contextual influences it undergoes in developing patho-physiology. In normal conditions, and even in low-grade epithelial tumors, TGFβ-activated Smads inhibit the transcription of cell-proliferative genes. In high-grade tumors, this inhibitory role is bypassed and TGFβ-sensitive tumor cells will activate sets of proliferative and pro-invasive genes. This switch seems dependent on genetic and epigenetic changes, and on parallel activation of other signaling cascades that co-operate with TGFβ signaling. This switch has been proposed to depend also on the tissue and on the stage of the disease. Alternatively, a total inhibition of TGFβ signaling is anticipated to result in diffused inflammation, autoimmunity and/or cardiac pathology, as shown by analysis in several gene knockout mouse models. Thus, the real challenge in pharmaceutical inhibition is to prevent excess TGFβ signaling whilst still maintaining a basal level in order to maintain e.g., in cancer its known early-phase inhibitory effects on tumor cell proliferation and tumor growth.

We suggest that the growing number of SIPs, including functionally and biochemically well-characterized SIP–TFs, provide new potential aptamers for interfering with high selectivity in ligand receptor-induced Smad activities in either TGFβ or BMP signaling, including in disease. Furthermore, many of these SIPs directly relate to disease as well, including cancer.

### 4.1. Zinc Finger TFs as SIPs: SIP1/Zeb2 and OAZ

#### 4.1.1. Smad-Interacting Protein-1 (Sip1, also Named Zeb2 and Zfhx1b)

**Figure 4:**
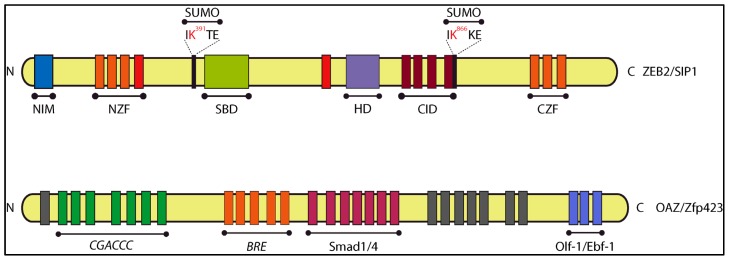
Schematic representation of Sip1 and OAZ domain structures. Sip1 contains two clusters of zinc fingers (NZF and CZF) that flank a centrally located homeodomain-like domain (HD). Between the HD and the CZF cluster, there is a segment containing 4 CtBP co-repressor interacting sequences (forming the CtBP-interacting domain, CID). Sip1 also binds the chromatin remodeling co-repressor NuRD. Sip1 is post-translationally modified by sumoylation (SUMO; exerted by Pc2). Sip1 contains a R-Smad-binding domain (SBD). OAZ contains 30 zinc fingers spread over clusters, mediating binding with DNA (to CGACC), a BMP–Smad-responsive element (BRE), Smad1/4 and Olf-1/Ebf-1 proteins (For more details, see main text).

Sip1 was identified in yeast two-hybrid screening using the MH2 domain of Smad1 as bait and a E12.5 mouse embryo cDNA library as source of prey fusion proteins [91]. Amongst the many candidate interacting prey proteins identified in this screening, a partial cDNA (encoding 626 aa) of a novel member of the small Zfhx1/Zeb zinc finger TF family was isolated. The full-length counterpart Sip1/Zeb2/Zfhx1b (1214 aa in human, 1215 aa in mouse) is, like the first identified member δEF1/Zeb1/Zfhx1a (with even more many alternative names), a DNA-binding TF. Zeb1 and Zeb2 share a similar protein domain organization (Figure 4). Zeb2, like Zeb1, acts mainly as repressor of transcription, but can also activate target gene transcription, depending on its co-factors, including in many BMP–Smad regulated processes [92,93]. Zeb2 modulates target gene transcription through binding with two zinc fingers in each of its two zinc finger clusters to a separated repeat of CACCT(G)—or in fewer cases CACANNT(G)—in regulatory regions of genes [94]. In BMP-treated oligodendrocyte progenitor cells Zeb2 binding to R-Smad–Smad4 complexes turns BMP–Smad-activated transcription of BMP-responsive inhibitory genes for myelination into repression, thereby stimulating myelination. However, Zeb2 achieves this also in part its direct activation of *Smad7*, encoding an inhibitory (anti-BMP) Smad [92]. Hence, Zeb2 is a BMP-inhibitory TF in this cell differentiation process, and likely other such processes, *in vivo*. Zeb1 and Zeb2 bind to CtBP co-repressors [95] and the chromatin-remodeling/co-repressor complex NuRD [96], and both full-length Zeb1 and Zeb2 can activate transcription by binding to P300/PCAF [97] (Figure 4). Zeb1 and Zeb2 levels themselves are under control of miRs, including in EMT, which underlies the invasive properties of epithelial-derived tumor cells [98–101]. Similar control circuits involving both Zeb proteins operate in tumor-initiating cells [102]. High levels of Zeb1 and/or Zeb2 in various cancers accompany bad prognosis.

Both proteins contain also a POU-like homeodomain (HD) that, based on amino acid changes as compared to cognate DNA-binding HDs, does not bind to DNA, unlike the true HD present in the Zeb-related *Drosophila* zinc finger protein Zfh1 [103], which likely has a dual mode of DNA-binding, *i.e.*, via the zinc fingers and via the HD. The HD-like domain is well conserved between Sip1 and δEF1 proteins. Only Zeb2 is a strong SIP. It binds to phospho–Smads via its 51 aa-long SBD; Zeb2 binds to the MH2 domain of TGFβ/Activin phospho-Smads2/3 and BMP phospho–Smads1/5/8 in the nucleus of ligand-stimulated cells. However, a number of Zeb2’s functions may be Smad-independent, underpinning its multiple modes of action (for a discussion, see [93]). While the binding between Zeb2 and R-Smads is direct, biochemical interaction studies with Zeb1 suggest that it lacks such SBD. Zeb1 interaction with activated Smads in various assays is very weak; it probably occurs indirectly, mediated by P300, at least when tested in overexpression experiments.

Mutations in *ZFHX1B* (chr2q22) cause Mowat–Wilson syndrome (MWS), a single-gene disorder (MIM#235730) characterized by various malformations that do not all appear in every patient. The major defects occur in the central nervous system and cause intellectual disability, absence of corpus callosum, microcephaly, epilepsy, and delayed motor development. These combine with neural crest defects (leading to craniofacial abnormalities in all MWS patients and Hirschsprung disease in most patients) and with a heterogeneous spectrum of other defects [104–107]. Haplo-insufficiency has been postulated as the major cause of the wide variety of symptoms in severe and milder forms of MWS.

Recently, a set of Trx-based peptide aptamers containing the entire Zeb2 SBD or *N*- or *C*-terminal deletions thereof, enabled us to re-define the SBD as a 14aa-long shorter linear sequence within the SBD, serving binding with both TGFβ/Activin/Nodal and BMP Smads. This interaction occurs only in cells stimulated with the respective ligands [108]. Furthermore, subtle mutation of residues within this 14aa-long SBD in a full-length Zeb2 enabled us to construct a Zeb2 variant Sip1 that no longer binds to Smads but in which the other known functional domains, including those for DNA binding, remain intact. This variant, whose construction was possible via the use of peptide aptamers, is a new tool to dissect Smad-dependent from Smad-independent functions of Zeb2, in particular in ligand-controlled processes, and for identifying accordingly SBD-dependent direct target genes, in stem cells, cancer cells and *in vivo* in knockout and knockin mice.

#### 4.1.2. OAZ (also named Zfp423)

While the zinc finger TF Zeb2 possesses a short, linear non-zinc-finger SBD, the multi-zinc finger TF OAZ/Zfp423 (with 30 zinc fingers in total) associates with Smad4 and the BMP–Smad Smad1 via its clustered zinc fingers 14–19, only in the presence of BMP2, while it binds DNA via a neighboring zinc finger cluster [109–111]. OAZ is also a binding partner for the bHLH-type TF Olf-1/Ebf-1 in the olfactory system and in lymphocyte development [112,113] (Figure 4). OAZ sequesters Olf-1/Ebf-1 in a hetero-meric complex, with accompanying failure to bind to the Olf-1/Ebf-1 binding sites on DNA. In the adult mouse, a number of marker genes characteristic for mature neurons are regulated by Olf-1/Ebf-1 in the olfactory system. OAZ is also present in neuroprogenitors and immature neurons in this system, in a complementary fashion to the mature marker OMP [112]. SELEX, using OAZ (as a fusion protein with glutathione-*S*-transferase, GST) and a DNA library of random 18-mers, allowed the identification of a palindromic consensus sequence composed of a half site GCACCC, a spacer of 2 bp (^A^/_T_^A^/_T_), and an inverted half-site GGGTGC; it also identified an imperfect inverted half-site with a single nucleotide substitution at one of the last two positions [113].

**Figure 5:**
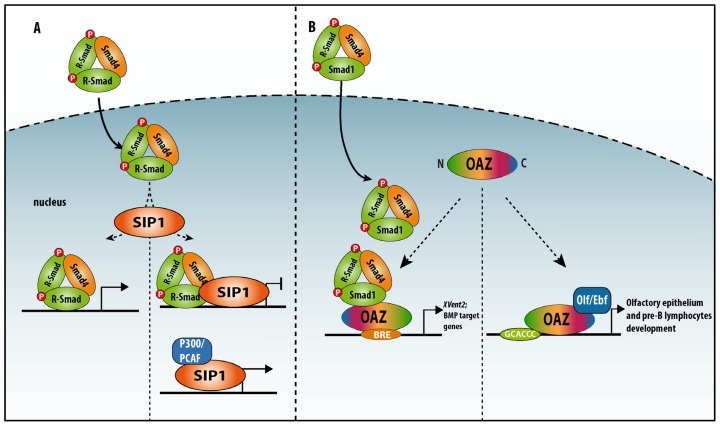
Action modes of Sip1 and OAZ in combination with Smads or other binding partners. (**A**) Sip1s binds to activated Smads and mediates repression of genes, including those that may be activated by the Smad complex that is unbound to Sip1. Sip1 can, however, also bind to P300/PCAF proteins, which turns Sip1 into a transcriptional activator; (**B**) OAZ binds to Smad 1/4 or Olf-1/Ebf-1 in a mutually exclusive manner. OAZ–Smad interaction results in activation of BRE-containing target promoter (e.g., *XVent1/2* in *Xenopus* embryos). When Olf/Ebf is bound at the OAZ *C*-terminal segment, OAZ no longer interacts with Smads, and *N*-terminal zinc finger cluster binds to a conserved palindromic sequence (for more details, see main text), stimulating transcription of genes involved in the olfactory epithelium and in B-lymphocyte development.

OAZ can bind the palindromic sequence via 7 zinc fingers, with a very low affinity for the half-site as monomer, while when as homodimer as well as heterodimer with Olf-1/Ebf-1 the affinity for this OAZ target DNA increases. Thus, the TF OAZ can form dimers on both the inverted and direct repeats, but not on the half-site. When in heterodimer complex with Olf-1/Ebl-1, OAZ eliminates transcriptional activation at the Olf-1/Ebf-1 binding site by sequestering the bHLH partner and preventing it from binding to DNA [113]. In *Xenopus*, OAZ binds to the promoter of *XVent2*, driving the expression of this gene during patterning of the lateral and ventral mesoderm. Activation of *XVent2* is strictly dependent on the binding of OAZ to BMP-activated Smad1. Therefore, OAZ is a SIP-TF and, interestingly, the binding of OAZ with phospho–Smad1 and Olf-1/Ebf-1 is mutually exclusive, and each complex binds to different promoters and other regulatory regions [109] (Figures 4 and 5B).

### 4.2. Non-TF SIPs

#### 4.2.1. Ski/SnoN

Ski/SnoN are negative regulators of TGFβ signaling that displace R-Smad–Smad4 complexes, thereby impairing TGFβ-induced cell proliferation arrest. Hence, it is not surprising that the expression of Ski/SnoN factors is found deregulated in several types of cancer. Furthermore, mutations in *SKI* were recently found to cause Sphrintzen–Goldberg syndrome with aortic aneurysm [114].

**Figure 6:**
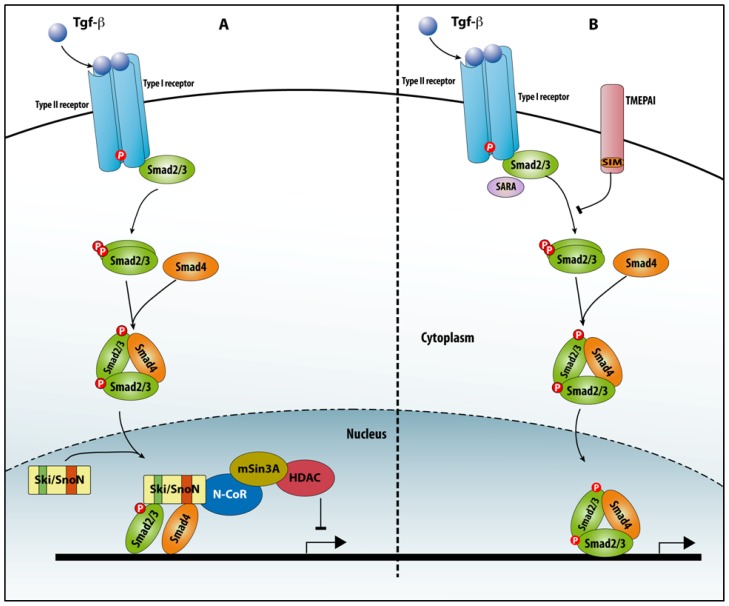
Schematic representation of Ski/SnoN and TMEPAI action modes. (**A**) Ski/SnoN acts as transcriptional repressor of Smad signaling. Ski/SnoN displaces the heterotrimeric complex R-Smads/Smad4 in the nucleus and binds to Smad2/3 and Smad4 via two distinct domains (green and orange in Ski/SnoN, respectively). Smads can still bind to DNA via their MH1 domain, and Ski/SnoN recruits mSin3A and HDAC for downregulating transcription; (**B**) The membrane protein TMEPAI competes with SARA for the binding to Smad2/3. TMEPAI possesses a Smad-interacting motif (SIM) in its intracytoplasmic domain, which sequesters Smad2 and Smad3 regardless of their phosphorylation status, thus preventing activation of Smad signaling.

Both Ski and Sno proteins have two specific, non-overlapping binding sites for Smad4 (named the SAND-like domain) and Smad2/3 (Figure 6A). The Ski/Smad4/R-Smad complex binds to DNA sequences via the MH1 domain of the Smads. Binding of Ski to Smad2/3 causes dissociation of P300 from the Smad complex, resulting in a reduction of P300-associated histone acetyltransferase (HAT) activity and recruitment of mSin3A and histone deacetylases (HDACs), which contributes to transcriptional downregulation [115]. Thus, in the case of Ski/SnoN, an efficient aptamer strategy would consist of initially targeting the binding sites for R–Smad or Smad4 and assess its potential to rescue e.g., TGFβ-induced cell proliferation arrest and perhaps apoptosis. Another route may be to document if the combination of two different aptamers, one directed against the Smad4 and one against the Smad2/3 binding region, is able to interfere with the effect(s) of TGFβ.

#### 4.2.2. TMEPAI

Transmembrane prostate androgen-induced (TMEPAI) is a mRNA induced by TGFβ and encodes a single-pass transmembrane protein. It negatively regulates Smad signalling: TMEPAI binds to TGFβ/Activin/Nodal Smads regardless of their activation status, via the Smad Interaction Motif (SIM) Pro–Pro–Asn–Arg, which in TMEPAI is similar to the SIM in the TFs Milk and Mixer [116] (Figure 6B). More studies, including experiments involving overproduced SARA–SBD-based aptamers, show that TMEPAI competes with SARA for the binding with Smads. Of interest, *TMEPAI* is overexpressed in patient specimens of breast, lung and prostate cancers. This makes TMEPAI an attractive new protein for aptamer studies in the field. Furthermore, such aptamers could be coupled with sequences that allow localization to the plasma membrane, e.g., via lipid modification, since only a small fraction of TMEPAI was found to co-localize with SARA in early endosomes.

### 4.3. Mutated Smads in Cancer and Other Diseases

TGFβ/BMP signaling is deregulated in cancers by various mechanisms, including mutations of R-Smad and Smad4 encoding genes. Recently, in a study comprising more than 700 primary colorectal cancer (CRC) tumors and 36 CRC cell lines, missense mutations have been mapped in the co-Smad encoding gene *SMAD4*, and in *SMAD2* and *SMAD3*[117]; these mutations cluster in two hot-spots located at the protein level in the MH2 domain of these Smads. In SMAD4 the first hot-spot is located between Asp351-Pro356 and map also to Arg361 (mapping to the L1 loop); the second series of hot-spot mutations affects Ala406, Arg515 and Lys428, which are involved in the binding of Smad4 to R-Smads. In SMAD2/3, again two mutational hot-spots have been observed: the first affects residues that are structurally homologous to those in (*i.e.*, L1 loop), the second affects the SSXS activation motif. Missense mutations in *SMAD3* have been observed to lead to aortic aneurysma, and this is also the case for other genes encoding components of the TGFβ family system [118–120]. We suggest that aptamers could be used to identify specific protein variants (as successfully demonstrated by Guida and co-workers [121] for mutant P53) associated with increased proliferation, tumorigenic potential and resistance to anti-cancer chemical compounds and tumorigenic potential. Indeed, these authors produced and characterized several peptide aptamers able to bind specifically to mutant P53, but not wild-type P53. These aptamers induced apoptosis only in P53 mutant positive cells. Similarly to what has been done for such P53 mutant, also the different missense Smad mutations could represent an important next target for aptamer studies.

## 5. Conclusions and Future Perspectives

Over the last 20 years, aptamers have been developed as novel tools to interfere with protein–protein interaction. Many nucleic acid aptamers and peptide aptamers, directed against various types of targets, initially often including proteins involved in EGFR signaling, have been selected, produced and validated. Their low cost, ease of production and high affinity are features that make aptamers tempting tools for basic research, as well as for future therapeutic purposes. Interestingly, as it has emerged that aptamers can be directed towards even a certain variant (*i.e.*, isoform or mutated) target protein, the targeting of the according specific variant-expressing cells—even in a mixed cell population—becomes conceptually possible.

Our increasing interest in aptamers comes from the successful use of aptamers for affecting growth factor receptor signal transduction, in particular hyper-activation of EGFR signaling in tumorigenesis and tumor progression. We feel that the time has come to identify aptamers that can interfere with signaling cascades exerted by Wnt and TGFβ family factors. These pathways are fine-tuned at different levels in various ways, including by protein–protein interactions, and the often multi-functional effector proteins on which these pathways converge (*i.e.*, β-catenin, Tcf-1/Lef-1 for Wnt; Smad proteins and SIPs for TGFβ) are not easy targets for a classical inhibitory treatment with small molecules. Moreover, a certain basal or homeostatic level of their signaling must often be maintained, making effective dosage with ligand-sequestering compounds or monoclonal antibodies very difficult. Aptamers provide also in such case a valuable alternative, including those that act at the level of the downstream-executing TFs or their complexes, whose composition and target gene dynamics start to be mapped intensively using various “omics” approaches.

## Figures and Tables

**Table 1:** Comparing aptamers with antibodies.

	Aptamers	Antibodies
Production	Chemical process carried out *in vitro* and at low cost standardized process	Requires, in most cases, an animal, increasing the cost activity; stability may vary from batch to batch
Targets	Any protein and any site, including toxin-specific targeting of mutant proteins, as well as of post-transcriptionally modified proteins or differentially expressed isoforms	Some epitopes difficult to target; toxins excluded as these are not tolerated by the animal; can target protein modifications, albeit often with low specificity and/or affinity
Selection/screening	Iterative *in vitro* selection procedure allows the obtaining of highly specific aptamers	Screening of large panels of (monoclonal) antibodies is fairly time-consuming and expensive
Modifications	Many chemical modifications available to increase stability or cellular uptake	Relatively few chemical modifications available
Immunogenicity	None reported that exceeds other antisense oligonucleotides or macromolecules	Proven immunogenicity, especially relevant to non-humanized antibodies

**Table 2:** Used scaffold proteins and modifications.

Scaffold	Structural element	Application	References	Remark
Thioredoxin A (TrxA)	1 loop	yeast two-hybrid phage display mammalian cells	[18,25]	
Staphylococcal nuclease	1 loop	functional screening	[21]	
Human Stefin A	3 sites	yeast two-hybrid	[26]	
Green Fluorescent Protein (GFP)	loop randomization	visual screening	[20]	
FKBP12-(peptide)-FRB-GST	fusion of three domains	screen for kinase inhibitors	[27]	ligand-regulated peptide aptamers (LiRPs; rapamycin)

**Table 3:** Aptamers used in traceable clinical trials.

NCT number	Target (aptamer + adjuvant)	Conditions	Phase/s	Funded by
NCT00950638	C5 complement (ARC1905 aptamer)	AMD	1	Industry
NCT00709527	C5 complement (ARC1905 aptamer + Lucentis)	AMD	1	Industry
NCT01089517	PDGF (E10030 aptamer + Lucentis)	AMD	2	Industry
NCT00569140	PDGF (E10030 aptamer)	AMD	1	Industry
NCT00312351	VEGF (Macugen)	Macular Degeneration	4	Industry
NCT00021736	VEGF	AMD and Choroidal Neovascularization	2/3	Industry
NCT00215670	VEGF (Macugen)	AMD	2/3	Industry
NCT00321997	VEGF (Macugen)	AMD	2/3	Industry
NCT00040313	VEGF (Macugen)	DME	2	Industry
NCT01487070	VEGF (Macugen)	Proliferative Diabetic Retinopathy	1	Other
NCT01487044	VEGF (Macugen)	DME		Other/Industry
NCT00632242	vWF (ARC1779)	Purpura Thrombotic, Thrombocytopenic von Willebrand Disease Type-2b	2	Industry
NCT01034410	Nucleolin (AS1411)	Acute Myeloid Leukemia	2	Industry
NCT01191372	BAX499 (ARC19499)	Hemophilia	1/2	Industry
NCT01194934	CXCL12/SDF-1 (NOX-A12)	Hematopoietic Stem Cell Transplantation	1	Industry
NCT00976378	CXCL12/SDF-1 (NOX-A12)	Autologous Stem Cell Transplantation	1	Industry/Other
NCT00976729	CCL2/MCP-1 (NOX-E36)	Chronic Inflammatory Diseases Type 2 Diabetes Mellitus Systemic Lupus Erythematosus	1	Industry
NCT00113997	REG1	Healthy	1	NIH
NCT00056199		von Hippel-Lindau Disease	1	NIH
